# Population pharmacokinetics and dose optimization of intravenous levofloxacin in hospitalized adult patients

**DOI:** 10.1038/s41598-022-12627-1

**Published:** 2022-05-27

**Authors:** Eko Setiawan, Mohd-Hafiz Abdul-Aziz, Menino Osbert Cotta, Susaniwati Susaniwati, Heru Cahjono, Ika Yunita Sari, Tjipto Wibowo, Ferdy Royland Marpaung, Jason A. Roberts

**Affiliations:** 1 Faculty of Medicine, University of Queensland Centre for Clinical Research (UQCCR), Level 8, Royal Brisbane and Women’s Hospital, The University of Queensland, Herston, Brisbane, QLD 4006 Australia; 2 Department of Clinical and Community Pharmacy, Center for Medicines Information and Pharmaceutical Care (CMIPC), Faculty of Pharmacy, University of Surabaya, 60293 Surabaya, East Java Indonesia; 3 Dr. Mohamad Soewandhie Public Hospital, 60142 Surabaya, East Java Indonesia; 4 PHC Hospital, 60165 Surabaya, East Java Indonesia; 5 Department of Clinical Pathology, Faculty of Medicine, University of Airlangga, Surabaya, East Java Indonesia; 6 Department of Pharmacy and Intensive Care Medicine, Royal Brisbane and Women’s Hospital, Brisbane, 4029 Australia; 7 Division of Anaesthesiology Critical Care Emergency and Pain Medicine, Nîmes University Hospital, University of Montpellier, 30029 Nîmes, France

**Keywords:** Infectious diseases, Drug therapy

## Abstract

Although levofloxacin has been used for the last 25 years, there are limited pharmacokinetic data to guide levofloxacin dosing in adult patients. This study aimed to develop a population pharmacokinetic model of levofloxacin for adult hospitalized patients and define dosing regimens that attain pharmacokinetic/pharmacodynamic target associated with maximum effectiveness. Blood samples were drawn from 26 patients during one dosing interval. Population pharmacokinetic modelling and dosign simulations were performed using Pmetrics®. Pathogen minimum inhibition concentration (MIC) distribution data from the European Committee on Antimicrobial Susceptibility Testing database was used to analyse fractional target attainment (FTA). A two-compartment model adequately described the data. The final model included estimated glomerular filtration rate (eGFR) to describe clearance. The population estimate for clearance was 1.12 L/h, while the volume of distribution in the central compartment and peripheral compartments were 27.6 L and 28.2 L, respectively. Our simulation demonstrated that an area under free concentration–time curve to MIC ≥ 80 was hardly achieved for pathogens with MIC ≥ 1 mg/L. Low FTA against *Pseudomonas aeruginosa* and *Streptococcus pneumoniae* were observed for patients with higher eGFR (≥ 80 mL/min/1.73m^2^). A daily levofloxacin dose of 1000 mg is suggested to maximise the likelihood of efficacy for adult patients.

## Introduction

Lower respiratory tract infections (LRTIs), including pneumonia, are a leading cause of death in many developing countries, including Indonesia^1^. Levofloxacin is a fluoroquinolone antibiotic and is considered a first-line empirical treatment for many patients with pneumonia^2–4^, given it has good coverage against Gram-negative, Gram-positive and atypical bacteria^5^. In addition, high concentrations of levofloxacin are achieved in lung tissue^6^. Nevertheless, resistance to levofloxacin has been documented and this may jeopardise its use against pathogens with reduced susceptibility^7^. Optimising levofloxacin dosing is an important strategy to overcome this problem.

Similar to other fluoroquinolones, the pharmacokinetic/pharmacodynamic (PK/PD) indices that best describe levofloxacin antibacterial activity are the area under the free concentration–time curve to MIC ratio (*f*AUC_0-24_/MIC) and the peak concentration to MIC ratio (C_max_/MIC)^8,9^. An *f*AUC_0–24_/MIC ≥ 80 has been recommended as a PK/PD target that is most likely to result in successful levofloxacin treatment^9^. Dosing regimens for levofloxacin should aim to achieve this PK/PD target, particularly in severely unwell patients.

An in-depth understanding of levofloxacin pharmacokinetics (PK), including PK variability and the covariate(s) influencing such variability, remains a key step in the design of dosing regimens that maximise the achievement of desired PK/PD targets. Previous data demonstrates differences in levofloxacin PK in patients^10–15^ compared with healthy subjects^16,17^. Additionally, levofloxacin population PK varies between ethnicities, with different PK parameters reported among Chinese^10,11^, Korean^12^, and Caucasian^13–15^ patients. Consequently, a standardised dose of levofloxacin may not always achieve comparable exposures across different ethnic groups of patients^10–13^.

Levofloxacin PK data in Indonesian hospitalized patients is not available and this may be problematic in optimising dosing in pneumonia, particularly in pathogens with higher MICs. Therefore, the aim of the present study was to describe the population PK of levofloxacin in adult Indonesian hospitalized patients and then to apply Monte Carlo simulation to define appropriate levofloxacin dosing regimens that can attain a priori PK/PD targets.

## Results

### Patient and sampling characteristics

Five data points from one ICU patient were considered biologically implausible and they were excluded for PK analysis resulting a total of 124 blood samples from 26 patients being potentially included in the final PK analysis. Almost all patients (88.5%) were diagnosed with pneumonia. No patient was given more than 750 mg of levofloxacin in a 24 h period. Table 1 describes the demographic characteristics of the patients. No concomitant therapy classified as known to significantly interact with levofloxacin PK was given in any of the study patients. No adverse drug reactions to levofloxacin were reported in any of the study patients.

**Table 1:** Demographic data of study participants.

Characteristic	Total patients (%)	ICU patients (%)	Non-ICU patients (%)
Total number	26	6	20
Male	16 (61.5)	3 (50.0)	13 (65.0)
With mechanical ventilation	4 (15.4)	4 (66.7)	0 (0)
Age (year)^a^	58.8 ± 16.4	62.5 ± 23.1	57.7 ± 14.5
Weight (kg)^a,b^	61.6 ± 12.1	63.0 ± 16.6	60.5 ± 7.82
Se_Cr_ (mg/dL)^a^	1.99 ± 1.48	2.03 ± 1.88	2.08 ± 1.56
Albumin (g/l)^a,c^	2.80 ± 0.50	3.13 ± 0.58	2.62 ± 0.26
eGFR_CKD-EPI_ (mL/min/1.73m^2^)^a^	52.7 ± 33.7	55.2 ± 40.7	52 ± 32.5
**Dose (once daily)**			
500 mg	8 (30.8)	2 (33.3)	6 (30.0)
750 mg	14 (53.9)	2 (33.3)	12 (60.0)
500–750*	1 (3.85)	0 (0)	1 (5.00)
750–500**	3 (11.5)	2 (33.3)	1 (5.00)

^a^Presented as mean ± SD.

^b^Number of patients with weight information: 5 (ICU patients) and 6 (non-ICU patients).

^c^Number of patients with albumin information: 3 (ICU patients) and 5 (non-ICU patients).

*Patient received two doses of 500 mg and then one dose of 750 mg.

**Two patients received one dose of 750 mg then a dose of 500 mg and another one patient received one dose of 750 mg then another two doses of 500 mg.

### Population PK model

Three concentration data points were further excluded from the population PK modeling due to possible contamination either with the previous (n = 2) or next dose (n = 1). An average of 4.77 blood samples per-patient (2–6 samples per patient) were obtained. Two blood samples per-patient were collected in two patients (7.69%). Levofloxacin was best described as a two-compartment model. The value of -2LL and AIC in the best structural model are described in Table 2.

**Table 2:** Estimates of levofloxacin pharmacokinetic parameters from the final model with covariate and model selection.

Parameter^a^	Mean	SD	CV%	Median	Shrink (%)
**Pharmacokinetic value**					
V_c_ (L)	27.6	19.1	69.3	26.4	3.71
CL (L/h)	1.12	0.58	52	0.90	1.60
Q (L/h)	30.9	16.4	53.2	33.2	5.81
V_p_ (L)	28.2	16.2	57.7	27.9	5.20

Compartment model	− 2LL	AIC			
**Model selection**					
One	393	399			
Two (with K_PC_–K_CP_)	272	283			
Two (with Q)	256	267			
Two (with Q and the additional of eGFR_CKD-EPI_ on CL) FINAL	250	261			

^a^*V*_*c*_ volume of central compartment, *CL* clearance, *Q* inter-compartment clearance, *V*_*p*_ volume of peripheral compartment, *KCP* the rate constant from the central compartment to the peripheral compartment, *KPC* the rate constant from the peripheral compartment to the central compartment, − *2LL* − 2*Log-likelihood at each cycle, *AIC* akaike information criterion at each cycle.

The only covariates that improved the goodness-of-fit of the scatter plots and decreased the value of -2LL and AIC significantly was eGFRCKD-EPI on CL (Table 2). The CL of levofloxacin was best described as the following equation: CL = (0.044* eGFR_CKD-EPI_) + 0.358, where CL is the levofloxacin CL and eGFR_CKD-EPI_ is the estimated glomerular filtration rate calculated with CKD-EPI equation.

**Figure 1:**
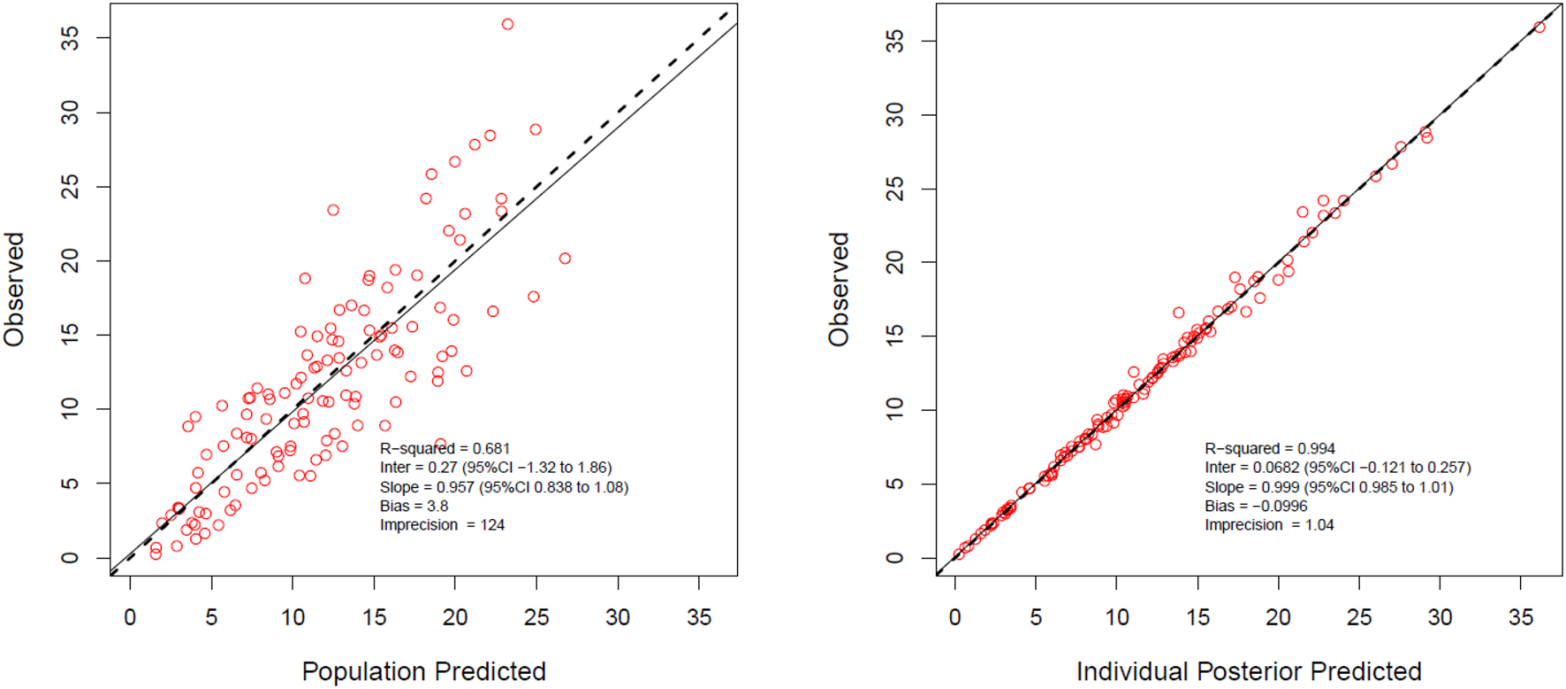
Diagnostic plot for the final covariate; (left) observed versus population predicted plasma concentrations and (right) individual predicted plasma concentrations.

**Figure 2:**
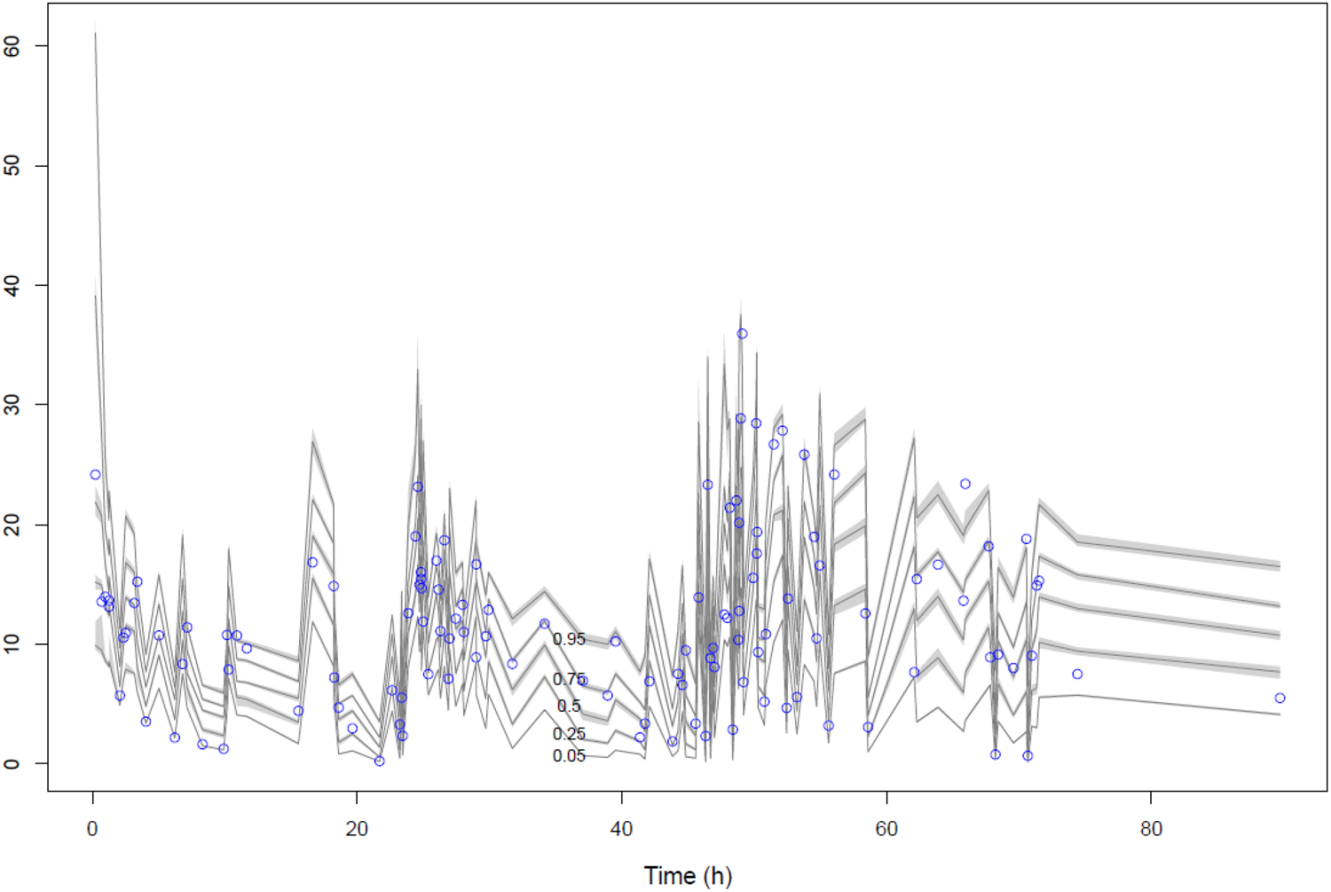
Visual predictive check plot of the final covariate two-compartment model; *y* axis indicated concentrations of levofloxacin (mg/L). Percentiles (with shaded 95% confidence interval) are the lines shown as 0.95, 0.75, 0.5, 0.25, and 0.05 values. Individual circles represent the observed concentration.

The goodness of fit of the observed versus predicted plots, both population, and individual prediction, for the final model with covariate were acceptable (Fig. 1). The distribution of the observed data within the percentiles of the simulated data is shown in Fig. 2. The estimated population PK parameters from the final model with covariate are presented in Table 2.

### Dosing simulation

#### PTA

**Figure 3:**
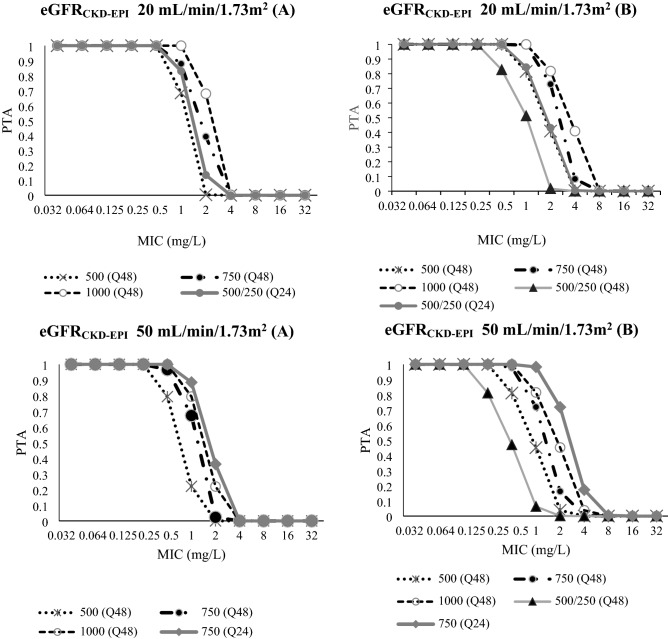
Probability of target attainment (PTA; *f*AUC/MIC ≥ 80) of several dosage regimens of levofloxacin at (**A**) the first 24-h and (**B**) steady state; for patients with eGFR_CKD-EPI_ 20 mL/min/1.73m^2^ and eGFR_CKD-EPI_ 50 mL/min/1.73m^2^; For the first dose (**A**) in both eGFR_CKD-EPI_ groups: the lines for levofloxacin 500/250 (Q48) and 750/500 (Q48) were relatively similar to 500 (Q48) and 750 (Q48), respectively; For the steady state (**B**) in both eGFR_CKD-EPI_: lines for 750/500 (Q48) was relatively similar to 500 (Q48). In eGFR_CKD-EPI_ 50 mL/min/1.72m^2^: the lines for levofloxacin 500/250 (Q24) and 500 (Q24) at the first dose (**A**) was relatively similar to 750 (Q48). While at steady state (**B**), the lines for levofloxacin 500/250 (Q24) and 500 (Q24) were relatively similar to 500 (Q48) and 1000 (Q48), respectively. To provide better clarity, lines with similarity shapes were not presented.

**Figure 4:**
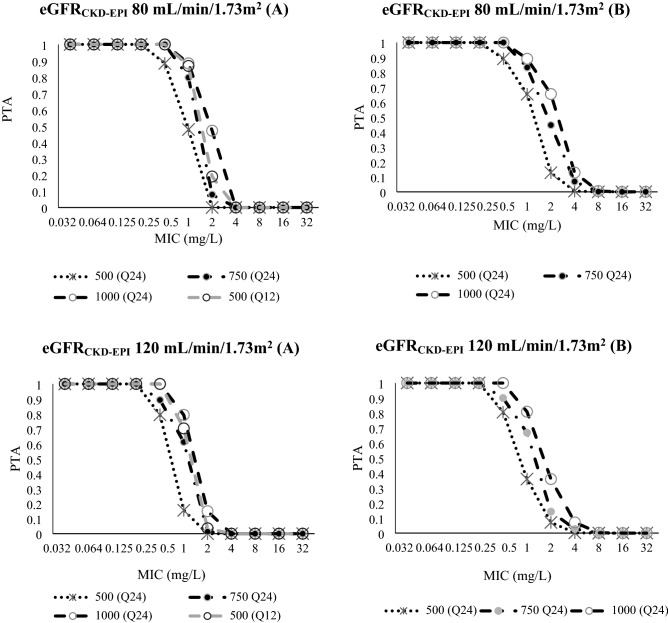
Probability of target attainment (PTA; *f*AUC/MIC ≥ 80) of several dosage regimens of levofloxacin at (**A**) the first 24-h and (**B**) steady state; for patients with eGFR_CKD-EPI_ 80 mL/min/1.73m^2^ and eGFR_CKD-EPI_ 120 mL/min/1.73m^2^; Dotted horizontal line represent 90% of PTA which considered as a successful or acceptable; For the steady state (**B**) in both CKD-EPI groups: the lines for levofloxacin 500 (Q12) was relatively similar to 1000 (Q24). To provide better clarity, lines with similarity shapes were not presented.

Figures 3 and 4 present the PTA of several dosing regimens of levofloxacin for several typical patient scenarios with eGFR_CKD-EPI_ level of 20–50 mL/min/1.73m^2^ and 80–120 mL/min/1.73m^2^, respectively. In all eGFR_CKD-EPI_ levels, there were various alternatives of dosing regimens observed that attained the PTA ≥ 90% against pathogens with MIC 0.5 mg/L even though not all resulted in a PTA ≥ 90% both on the first day of treatment and at a steady state condition. The highest MIC at which ≥ 90% PTA could be achieved among all dosing regimens is in the supplementary file (suppl. Table S1).

For patients with eGFR_CKD-EPI_ of 80 and 120 mL/min/1.73m^2^, a daily dose of 500 mg every 24 h has a PTA ≥ 90% against an MIC of 0.25 mg/L which is the epidemiological cut-off value (ECOFF) for *Escherichia coli* and *Klebsiella penumoniae*^18^. Increasing the dose to 750 mg every 24 h, ensures an acceptable PTA against an MIC value of 0.5 mg/L (the ECOFF for *Staphylococcus aureus*)^18^ for both the first day of treatment and at steady state for patients with eGFR_CKD-EPI_ 80 mL/min/1.73m^2^ (≥ 90% and ≥ 90%) and eGFR_CKD-EPI_ 120 mL/min/1.73m^2^ (89.3% and ≥ 90%).

In all eGFR_CKD-EPI_ levels and for all investigated dosing regimens, it was difficult to obtain a PTA ≥ 90% for an MIC ≥ 1 mg/L. Even the highest daily dose of levofloxacin, either given as 1000 mg every 24 h or 500 mg every 12 h, could not attain a PTA ≥ 90% against MIC ≥ 1 mg/L.

#### FTA

The FTA of all levofloxacin dosing regimens over four different eGFR_CKD-EPI_ levels achieved ≥ 90% against the empirical MIC distribution of *M. cattharalis* and *H. influenzae* (detailed percentages are not shown). The empirical FTAs were determined against *E. coli*, *K. pneumoniae, P. aeruginosa*, *S. aureus*, and *S. pneumoniae* for patients with eGFR_CKD-EPI_ 20–120 mL/min/1.73m^2^ (Table 3). While it can be found in each eGFR_CKD-EPI_ level group that some dosing regimens attained an FTA ≥ 90% against *E. coli*, *K. pneumoniae*, and *S. pneumoniae*, most levofloxacin dosing regimens only attained an FTA between 81 and 84% against *S. aureus*. Moreover, the FTAs of almost all of the simulated dosing regimens in all eGFR_CKD-EPI_ levels are far lower than the target of ≥ 90% against *P. aeruginosa.*

**Table 3:** FTA for various dosage regimens of levofloxacin against the empiric EUCAST MIC distributions of several Gram positive and Gram negative bacteria for patients with eGFR_CKD-EPI_ 20, 50, 80, 120 mL/min/1.73m^2^.

Levofloxacin dosage regimens	FTA (%) by bacteria and eGFR_CKD-EPI_ (mL/min/1.73m^2^)																			
	*E. coli*				*K. pneumoniae*				*P. aeruginosa*				*S. aureus*				*S. pneumoniae*			
	20		50		20		50		20		50		20		50		20		50	
	a	b	a	b	a	b	a	b	a	b	a	b	a	b	a	b	a	b	a	b
500 (Q48)	88	88	86	87	85	88	80	82	66	73	52	56	82	83	81	81	72	83	33	51
750 (Q48)	88	89	88	88	88	91	85	86	74	80	65	68	83	84	82	82	88	98	71	75
1000 (Q48)	89	89	88	88	90	92	87	88	79	84	70	73	83	87	83	83	98	98	81	83
500/250 (Q48)	88	87	86	85	85	83	80	76	66	58	52	35	82	81	81	73	72	56	33	14
750/500 (Q48)	88	88	88	87	88	88	85	83	74	74	65	57	83	83	82	81	88	84	71	52
500/250 (Q24)	88	88	88	87	87	88	85	83	70	73	65	57	82	83	82	81	83	85	71	52
500 (Q24)	-	-	88	88	-	-	85	88	-	-	65	74	-	-	82	83	-	-	71	85
750 (Q24)	-	-	88	89	-	-	88	91	-	-	73	80	-	-	83	85	-	-	88	97

Levofloxacin dosage regimens	FTA (%) by bacteria and eGFR_CKD-EPI_ (mL/min/1.73m^2^)																			
	*E. coli*				*K. pneumoniae*				*P. aeruginosa*				*S. aureus*				*S. pneumoniae*			
	80		120		80		120		80		120		80		120		80		120	
	a	b	a	b	a	b	a	b	a	b	a	b	a	b	a	b	a	b	a	b
500 (Q24)	87	87	86	87	83	85	80	82	59	63	50	55	81	82	80	81	54	68	28	44
750 (Q24)	88	88	87	87	87	88	84	85	68	74	61	64	82	84	81	82	81	85	64	69
1000 (Q24)	88	89	88	88	89	90	87	88	75	78	69	73	83	84	82	83	88	90	81	82
500 (Q12)	88	89	88	88	87	90	86	87	71	77	66	72	83	84	82	83	86	89	73	80

– not simulated, *a* simulation at the first dose, *b* simulation at the steady state, *Q48* given every 48 h, *Q24* given every 24 h, *Q12* given every 12 h.

Considering the MIC distribution of the susceptible strains, an acceptable FTA (≥ 90%) can be found in all different groups of eGFR_CKD-EPI_ levels by giving any dosage regimen against *E. coli*, *K.pneumoniae, H. influenzae*, *M. cattharalis*, and *S. aureus* (detailed percentages for each pathogen are not shown). A dose of 1000 mg every 24 h is most likely to achieve desirable FTA for both *P. aeruginosa* and *S. pneumoniae* in patients with eGFR_CKD-EPI_ ≥ 50 mL/min/1.73m^2^ (Table 4).

**Table 4:** FTA for various dosage regimens of levofloxacin against the directed EUCAST MIC distributions of several gram positive and negative bacteria for patients with eGFR_CKD-EPI_ 20, 50, 80, and 120 mL/min/1.73m^2^.

Levofloxacin dosage regimens	FTA (%) by bacteria and eGFR_CKD-EPI_ (mL/min/1.73m^2^)															
	*P. aeruginosa*								*S. pneumoniae*							
	20		50		80		120		20		50		80		120	
	a	b	a	b	a	b	a	b	a	b	a	b	a	b	a	b
500 (Q48)	79	88	62	68	–	–	–	–	73	84	33	51	–	–	–	–
750 (Q48)	89	96	78	82	–	–	–	–	89	99	71	7	–	–	–	–
1000 (Q48)	95	97	85	88	–	–	–	–	99	99	82	84	–	–	–	–
500/250 (Q48)	79	69	62	42	–	–	–	–	73	56	33	14	–	–	–	–
750/500 (Q48)	89	89	78	69	–	–	–	–	89	85	72	52	–	–	–	–
500/250 (Q24)	84	89	79	69	–	–	–	–	84	86	72	52	–	–	–	–
500 (Q24)	–	–	79	89	71	76	65	72	–	–	72	85	54	69	40	58
750 (Q24)	–	–	89	96	83	89	80	84	–	–	89	98	82	85	73	79
1000 (Q24)	–	–	–	–	90	93	87	90	–	–	–	–	89	91	86	88
500 (Q12)	–	–	–	–	86	93	82	90	–	–	–	–	87	90	80	86

– not simulated, *a* simulation at the first dose, *b* simulation at the steady state, *Q48* given every 48 h, *Q24* given every 24 h, *Q12* given every 12 h.

## Discussion

To the best of our knowledge, ours is the first study to describe the population PK of levofloxacin in Indonesian hospitalized patients. In our study, we combined ICU and non-ICU patients into one group to increase the generalisability of the results. There was an non-statistically significant difference in levofloxacin PK observed in our study when compared to previous published data^13^. Our dosing simulation indicated that higher than 750 mg daily dose of levofloxacin may be required to achieve *f*AUC/MIC ≥ 80 in patients with eGFR_CKD-EPI_ values ≥ 80 mL/min/1.73m^2^ and for infections with an MIC ≥ 1 mg/L.

Our PK model is consistent with previously published articles describing levofloxacin PK using a two-compartment model^12–15^. The value of estimated PK parameters, however, are relatively different compared with other studies. For example, we found that the CL of levofloxacin in Indonesian hospitalized patients is relatively lower (CL = 1.12 L/h) compared to what has been reported in other studies including Italian patients (CL = 8.66 L/h); Korean patients (CL = 6.19 L/h); and patients from the United States (9.27 L/h)^12,13,15^. Given the significant influence of renal function on levofloxacin CL^12–15^, the lower levofloxacin CL in our study could potentially be related to the lower renal function associated with this patient cohort. The mean (± SD) eGFR of patients in our study was 53 (± 33) mL/min/1.73m^2^ while the mean creatinine clearance (CL_Cr_ ) reported in the study by Roberts et al*.*, Kiem et al*.,* and Preston et al*.* were 70 (± 67; in critically ill group) and 70 (± 32; in non-critically ill group), 80.6 (± 28.2) mL/min, 82.9 (± 31.6), respectively^12,13,15^. It is worth noting, however, that renal function might not be sufficient to completely describe the variability of levofloxacin CL. In our model, renal function might contribute to explain around 52% of CL variability, while it was found to be 45% and 14% in models developed by Roberts et al. and Kiem et al., respectively^12,13^. Given renal CL represents approximately 60% of total body CL of levofloxacin, it is understandable that renal function alone cannot explain CL variability^19^. Therefore, it is likely that the different reported CL value between our model and other reported models might also be influenced by non-renal CL.

Nevertheless, the value of eGFR or CL_Cr_ is likely to be the foundation to optimise dosing of levofloxacin. Altered dosing approaches should be implemented in patients depending on their eGFR_CKD-EPI_ values, especially if a causative organism with MIC > 0.5 mg/L is a concern. Our dosing simulation indicates that the majority of the simulated dosing regimens in all eGFR_CKD-EPI_ groups could attain PTA ≥ 90% against an MIC of ≤ 0.5 mg/L. This PTA is, more difficult to be attained against an MIC of ≥ 1 mg/L, especially in patients with higher eGFR_CKD-EPI_ levels. For an MIC 1 mg/L, our study found that every alternate day dosing regimen with 750 or 500 mg every 48 h might still be appropriate for patients with eGFR_CKD-EPI_ 20 mL/min/1.73m^2^ should a PTA of around 85% be clinically acceptable at both the first day of treatment and steady state. A daily dosing regimen, however, should be implemented for patients with eGFR_CKD-EPI_ ≥ 50 mL/min/1.73m^2^ and this supports product information recommendations^20^. It is worth mentioning that the highest dose of levofloxacin commonly prescribed at the research sites of our study, i.e., 750 mg every 24 h, could obtain acceptable PTA against MIC of 1 mg/L at the fifth day of treatment for patients with eGFR_CKD-EPI_ 50 mL/min/1.73m^2^ but not for patients with a eGFR_CKD-EPI_ of 80 mL/min/1.73m^2^ or 120 mL/min/1.73m^2^. Maintaining a dose of 750 mg makes does not resolve the possibility of underexposure of levofloxacin at higher eGFRs. However, it should be anticipated that eGFR or CL_Cr_ may not always be available in the Indonesian hospital setting at the time when doses are initiated or dosing adjustments made^21^. Serum creatinine is more widely reported and so it is likely that dosing adjustments are made based on this reported value. Notably, Se_Cr_ may not accurately determine renal function^22^, and so this may limit the effort to optimise levofloxacin exposure.

Doses higher than 750 mg daily may be required to achieve *f*AUC/MIC ≥ 80, particularly for patients with higher eGFR_CKD-EPI_ values and for infections with an MIC ≥ 1 mg/L. Our simulations emphasize that a daily dose of 1,000 mg levofloxacin could attain a PTA slightly below 90% for patients with eGFR_CKD-EPI_ 80 mL/min/1.73m^2^. Roberts et al., however, suggested that this higher daily dose could not attain a PTA ≥ 60% in patients with CL_Cr_ 70 mL/min^13^. A higher model estimated population value for levofloxacin CL compared to what we found in our study is the likely explanation for this relatively lower reported PTA^13^. However, for patients with eGFR_CKD-EPI_ higher than 80 mL/min/1.73m^2^, our highest simulated dosing regimen could not provide adequate PTA against pathogens with an MIC ≥ 1 mg/L.

Given the MIC susceptibility profile and therefore more easily attainable PK/PD targets, we have demonstrated that a variety of levofloxacin dosing regimens can potentially achieve relatively optimal FTA against *H. influenzae M. cattarhalis*, *E. coli*, *K. pneumoniae*. The borderline FTA (around 85%) could be potentially attainable against *S. aureus*. Of the pathogens studied, effective levofloxacin exposure against *P. aeruginosa* and *S. pneumoniae* are concerning. A high percentage of *P. aeruginosa* (45.7%) and *S. pneumoniae* (79.9%) in the EUCAST’s MIC distribution data had MIC ≥ 1 mg/L and this may contribute to the reported lower FTA attainment^18^. This may indicate that levofloxacin may not be a good choice to be used as an empirical therapy for both ICU and non-ICU patients where *P. aeruginosa* and *S. pneumoniae* are predominantly reported in Indonesian hospitals. Nevertheless, our study highlights the potential of using levofloxacin as targeted therapy in infections caused by susceptible strains of *P. aeruginosa* and *S. pneumoniae*. A dose of 750 mg every 24 h and 1,000 mg every 24 h may provide adequate exposure for Indonesian patients with eGFR_CKD-EPI_ 50 mL/min/1.73m^2^ and ≥ 80 mL/min/1.73m^2^. Combination with other antibiotics or other broader antibiotics may be considered at the time when MIC is not known and then de-escalation to levofloxacin monotherapy with appropriately adjusted dosing after MIC is in hand. Furthermore, with the increasing number of LRTIs caused by *Stenotrophomonas maltophilia* particularly in Asia–Pacific region, dosing simulations against the MIC distribution of this biofilm-forming bacterium could be considered as an important area for future research^23^.

It should be noted that there are some limitations in our study. First, we could not collect all blood samples within the precise time specifications as originally planned due to some technical challenges. These unstructured blood samplings are prone to cause imprecision in estimating the PK of levofloxacin. However, by having a mean of 4 to 5 blood samples per-patient and employing a population PK approach with Bayesian priors, we believe that our analysis provides a reasonable population PK estimation. Second, our PK model may not truly describe the distribution of levofloxacin PKs in hospitalized Indonesian patients given that only 26 patients were included in the study. We performed Monte Carlo simulation to virtually enlarge the sample sizes and predicted the probability of several levofloxacin dosing regimens to attain adequate PD exposure. In the settings where the estimated PKs from a substantial number of sample size are unavailable, Monte Carlo simulation could be considered as a rational approach to identify the achievement of PK/PD exposure from varied dosing regimens. Third, even though levofloxacin in our study was given predominantly to patients with pneumonia, we could not measure the concentration of levofloxacin at the site of infection. Given that PK/PD indices are most commonly based on blood exposures of drug and that levofloxacin extensively penetrates into the epithelial lining fluid^6^, we believe that our findings are translatable. Fourth, we used MIC data from EUCAST database in our dosing simulations and these may not represent the MIC distributions in Indonesian settings. Therefore, caution should be taken when extrapolating these results to Indonesian hospitals, with the recommendation being that each facility consider local antibiogram data when making therapeutic and dosing decisions involving levofloxacin. It is worth mentioning though that susceptibility surveillance is not adequately conducted in every hospital setting in developing countries such as Indonesia^24,25^. Finally, although we included patients with eGFR_CKD-EPI_ ≤ 20 mL/min/1.73m^2^, our findings might not be applicable for those with renal replacement therapy (RRT) as they were excluded from our study.

## Conclusions

We have described the PK of levofloxacin in Indonesian hospitalized patients. Changes in CL of levofloxacin in our study was significantly influenced by changes in eGFR_CKD-EPI_ and dose adjustment should be made accordingly. Our simulations found that an acceptable PTA (≥ 90%) could be obtained against a MIC of ≤ 0.5 mg/L among all simulated eGFR_CKD-EPI_ values. While for an MIC of ≥ 1 mg/L, a PTA of 90% is likely difficult to attain, in particular among those patients with eGFR_CKD-EPI_ of 120 mL/min/1.73m^2^. Higher doses of levofloxacin provide adequate coverage against majority LRTI pathogens, however, high doses are needed to achieve acceptable FTAs against *P. aeruginosa* and *S. pneumoniae.*

## Methods

### Study design and setting

A prospective observational PK study was conducted in two Indonesian hospitals from November 2018 to November 2019. Patients aged ≥ 18 years old admitted to the intensive care unit (ICU) and non-ICU wards and receiving intravenous levofloxacin were included in this study. Patients with a plan for RRT or extracorporeal membrane oxygenation (ECMO) at the time of sampling and/or known to be pregnant were excluded. The study protocol was approved by the Ethics Committee of Dr. Ramelan Navy Hospital (approval number 76/EC/KERS/2019) and The University of Queensland Human Research Ethics Committee (approval number 2018001592). Written informed consent from the patient or legal substitute decision-maker was obtained prior to sampling.

### Drug administration, sampling procedure, and data collection

Levofloxacin was administered as a 30-min intermittent infusion and the dosing regimens were at the discretion of the treating team. The aim was to obtain six blood samples (each sample: 3 mL, using lithium heparin as an anticoagulant) per-patient after the administration of 500 or 750 mg of levofloxacin intravenously during one dosing interval. All blood specimens were immediately centrifuged for 15 min at 3000 rpm after sampling and the aliquots were immediately frozen at − 20 °C. All frozen aliquots were further moved to − 80 °C within one week after the centrifugation.

The dose given, time of administration, number of blood samples and number of prior levofloxacin doses were recorded. Demographic data, including gender, age, body weight, and laboratory data, including serum creatinine (Se_Cr_) and albumin, were collected from patients’ medical records. The estimated glomerular filtration rate (eGFR) was calculated using the Chronic Kidney Disease Epidemiology Collaboration (CKD-EPI) equation^26^. For ICU patients, the use of mechanical ventilation was recorded. All data were recorded on the day of recruitment. All medications administered concomittantly with the levofloxacin were recorded and further screened for potential interactions with levofloxacin^27^. Interactions classified as “avoid combination” and “usually avoid combination” in the reference used in our study were considered as clinically relevant interactions^27^.

### Bioanalytical methods

Determination of levofloxacin concentrations in plasma was performed by a validated ultra-high performance liquid chromatography with tandem mass spectrometry (UHPLC-MS/MS) method on a Nexera liquid chromatograph connected to a 8030 + triple quadrupole mass spectrometer (Shimadzu, Kyoto, Japan). Test samples were assayed in batches alongside calibrators and quality controls, and results were subject to batch acceptance criteria. Sample (10 μL) was spiked with internal standard (ciprofloxacin) and protein was precipitated using acetonitrile. An aliquot of 0.2 μL of the supernatant was injected onto the UHPLC-MS/MS instrument. The stationary phase was a Kinetex C8 100 × 2.1 mm (1.7 µm) analytical column preceded by a SecurityGuard-Ultra C8 guard cartridge (Phenomenex, Torrence, USA). Mobile phase A was 0.2% formic acid in water (v/v), and mobile phase B was 0.2% formic acid in acetonitrile (v/v). Separations were effected with a gradient from 10 to 80% of mobile phase B at a flow of 0.3 mL/min, producing back pressure of approximately 3600 psi. Levofloxacin was monitored in positive mode at the mass-to-charge ratio (m/z) of 362.0 → 318.1. The standard internal ciprofloxacin was monitored in positive mode at m/z of 332.2 → 314.1. The assay method was linear from 0.1 to 50 mg/L (precision of 10.2% and accuracy of − 4.2% at LLOQ of 0.1 mg/L). Precision of 5.9, 2.1, and 5.8% and accuracy of 6.4, 0.9, and − 4.7% at levofloxacin concentrations of 0.3, 2 and 40 mg/L. The precision and accuracy of LLOQ and QCs and the matrix effect validation met the US FDA guidelines^28^.

### Population pharmacokinetic data analysis

Levofloxacin plasma concentration–time data were fitted to generate the population PK model using non-parametric adaptive grid (NPAG) algorithm in Pmetrics® software (version 1.9; Laboratory of Applied Pharmacokinetics and Bioinformatics, Los Angeles, CA, USA) for R 3.41^29^. Classic one-compartment and two-compartment models, with intercompartmental distribution, represented either as K_PC_–K_CP_ or Q were initially evaluated as a potential PK model. First-order processes were used to describe the elimination of levofloxacin from the central compartment and inter-compartmental distribution in two-compartment model. Both lambda (ranging from 0.1 to 0.9) and gamma (ranging from 1 to 9) error models were tested for each PK model.

The following were evaluated as potential covariates for volume of distribution (V_d_) and clearance (CL) of levofloxacin using linear, exponential regression and power (using population-median-normalised and allometric) model: gender, Se_Cr_, hospitalisation type (ICU and non-ICU), mechanical ventilation, and eGFR_CKD-EPI_. The value of 0.75 was used as a coefficient in the allometric model for age, Se_Cr_, and eGFR_CKD-EPI_. The final estimated PK parameters are presented as mean, standard deviation (SD), percentage coefficient of variation (%CV), and median value. The %CV was used to describe inter-individual PK variability.

### PK model diagnostics

The goodness of fit of the PK model was assessed using inspection of observed versus predicted plots, both population and individual predictions. The following indicators were used to identify the best structural and error model: (1) improvement of the scatterplot, (2) improvement of the intercept (close to 0) and slope (close to 1) of linear regression, (3) an increased value of the coefficient of determination (r^2^; close to 1) of the linear regression. In addition to this, a statistical reduction of − 2 log-likelihood (− 2LL; a decrease value of 3.84 corresponds to P < 0.05) and the lowest value of Akaike Information Criteria (AIC) scores were also used to choose the best structural and error model. Once the structural model was chosen, each covariate was separately added to that particular model. Only covariates that could improve the scatterplot, r^2^, intercept, slope, and a statistically significant improvement of − 2LL and AIC was retained in the final model. The internal validation of the final model with covariates was assessed by a visual predictive check (VPC) with 1000 simulations. The distribution of the observed concentration in this simulation was plotted and visually examined.

### Dosing simulations

Monte Carlo simulations (n = 1000) were undertaken using Pmetrics® to identify the probability of target attainment (PTA) of achieving the a priori PK/PD target both at first 24-h and at the fifth day of levofloxacin treatment (steady state) against a specific MIC value ranging from 0.004 to 512 mg/L. The a priori PK/PD target was *f*AUC_0-24_/MIC ≥ 80 and the protein binding of levofloxacin was set at 30%^9,30^. The simulations were conducted in four different eGFR_CKD-EPI_ levels, including 20, 50, 80, and 120 mL/min/1.73m^2^. The standard dosing regimens of levofloxacin for each eGFR_CKD-EPI_ level were simulated, including 500 mg i.v. followed by 250 mg every 48 h, 750 mg followed by 500 mg every 48 h, 500 mg followed by 250 mg every 24 h, 500 mg every 48 h, and 750 mg every 48 h (for eGFR_CKD-EPI_ 20 and 50 mL/min/1.73m^2^); 500 mg every 24 h and 750 mg every 24 h (eGFR_CKD-EPI_ 50, 80, and 120 mL/min/1.73m^2^)^20,31^. In addition, intensified dosing regimens up to the highest safe daily dose found in the literature (1000 mg/day) were also simulated for each eGFR_CKD-EPI_ level group, including 1000 mg every 48 h (for eGFR_CKD-EPI_ 20 and 50 mL/min/1.73m^2^); 500 mg every 12 h and 1000 mg every 24 h (eGFR_CKD-EPI_ 80, and 120 mL/min/1.73m^2^)^32^. All PTA simulations were assessed on the first day of treatment (first 24 h) and at steady state (defined as the fifth day of treatment). A PTA value ≥ 90% for a particular MIC value was considered optimal in our study.

To calculate the fractional target attainment (FTA), the PTA of each dosing regimen was compared against the MIC distribution of pathogens, commonly causative of LRTIs, obtained from the European Committee on Antimicrobial Susceptibility Testing (EUCAST) database (available from www.eucast.org; accessed 01 August 2020)^18^. The empirical FTA was calculated considering the whole range of MIC distribution of *Eschericia coli* (n = 9144)*, Klebsiella pneumoniae* (n = 3674)*, Haemophillus influenzae* (n = 22,910), *Pseudomonas aeruginosa* (n = 14,871), *Moraxella cattharalis* (n = 5259)*, Staphylococcus aureus* (n = 27,556), *Streptococcus pneumoniae* (n = 85,564). While the directed FTA was calculated by considering a range of MICs of the susceptible strains of each pathogen. Any dosage regimen that achieved the acceptable FTA (i.e. ≥ 90%) was considered a successful dosage regimen either for directed or empirical levofloxacin therapy.

### Statistical analysis

Descriptive analysis using frequencies (%) for categorical data and mean (± standard deviation; SD) for continuous data in the demographic of patients were conducted using Microsoft Excel v2016.

### Ethics approval

Approval was obtained from Ethics Committee of Dr. Ramelan Navy Hospital (approval number 76/EC/KERS/2019) and The University of Queensland Human Research Ethics Committee (approval number 2018001592). The procedures used in this study adhere to the tenets of the Declaration of Helsinki.

### Consent to participate and for publication

Written informed consent from the patient or legal substitute decision-maker was obtained prior to sampling.

## Supplementary Information

Supplementary Table 1.

## Data Availability

The datasets generated during and/or analysed during the current study are available from the corresponding author on reasonable request.
