# Supplementary material for: Population pharmacokinetics and dose optimization of intravenous levofloxacin in hospitalized adult patients
Source: Sci Rep. 2022 May 27;12:8930. doi: 10.1038/s41598-022-12627-1 (PMC9142570; doi:10.1038/s41598-022-12627-1)
Supplement: Supplementary file 1 — Supplementary Table 1. [file 41598_2022_12627_MOESM1_ESM.docx]

**Title of Manuscript**

**Population pharmacokinetics and dose optimization of intravenous levofloxacin in hospitalized adult patients**

Eko Setiawan^a,b^, Mohd-Hafiz Abdul-Aziz^a^, Menino Osbert Cotta^a,^*, Susaniwati Susaniwati^c^, Heru Cahjono^c^, Ika Yunita Sari^c^, Tjipto Wibowo^d^, Ferdy Royland Marpaung^d,e^, Jason A. Roberts^a,f,g^

^a^University of Queensland Centre for Clinical Research (UQCCR), Faculty of Medicine, The University of Queensland, Brisbane 4006, Australia

^b^Department of Clinical and Community Pharmacy, Center for Medicines Information and Pharmaceutical Care (CMIPC), Faculty of Pharmacy, University of Surabaya, 60293, Surabaya, East Java, Indonesia

^c^dr. Mohamad Soewandhie Public Hospital, 60142, Surabaya, East Java, Indonesia

^d^PHC Hospital, 60165, Surabaya, East Java, Indonesia

^e^Department of Clinical Pathology, Faculty of Medicine, University of Airlangga, Surabaya, East Java, Indonesia

^f^Departments of Pharmacy and Intensive Care Medicine, Royal Brisbane and Women’s Hospital, Brisbane 4029, Australia

^g^Division of Anaesthesiology Critical Care Emergency and Pain Medicine, Nîmes University Hospital, University of Montpellier, Nîmes 30029, France

*Address correspondence to:

Menino Osbert Cotta

E-mail address: [m.o.cotta@uq.edu.au](mailto:m.o.cotta@uq.edu.au)

Phone number: +61-7334 61814

Postal address:

Level 8, UQ Centre for Clinical Research (UQCCR)

Royal Brisbane and Women's Hospital

Herston Qld 4029

**Supplementary files**

**Suppl. Tabel 1.** The MIC breakpoints attaining *f*AUC_0–24_/MIC ≥ 80 for levofloxacin dosing regimens in four different eGFR_CKD-EPI_ levels

| Dosing regimen | MIC breakpoints (mg/L)* | | | |
| --- | --- | --- | --- | --- |
|  | **20 mL/min/1.73m^2^** | **50 mL/min/1.73m^2^** | **80 mL/min/1.73m^2^** | **120 mL/min/1.73m^2^** |
| 500 (Q48) | 0.5/0.5 | 0.25/0.25 | - | - |
| 750 (Q48) | 0.5/1 | 0.5/0.5 | - | - |
| 1000 (Q48) | 1/1 | 0.5/0.5 | - | - |
| 500/250 (Q48) | 0.5/0.25 | 0.25/0.125 | - | - |
| 750/500 (Q48) | 0.5/0.5 | 0.5/0.25 | - | - |
| 500/250 (Q24) | 0.5/0.5 | 0.5/0.25 | - | - |
| 500 (Q24) | - | 0.5/0.5 | 0.25/0.25 | 0.25/0.25 |
| 750 (Q24) | - | 0.5/1 | 0.5/0.5 | 0.25/0.5 |
| 1000 (Q24) | - | - | 0.5/0.5 | 0.5/0.5 |
| 500 (Q12) | - | - | 0.5/0.5 | 0.5/0.5 |

Note Supplementary Table 1:

*The highest MIC at which the probability of target attainment (PTA) ≥ 90%

The first value presented in each PK/PD breakpoint represented the highest MIC at which PTA ≥ 90% for the first and fifth administration, respectively.

-, not simulated
